# Effects of a history of headache and migraine treatment on baseline neurocognitive function in young athletes

**DOI:** 10.1186/s10194-022-01432-w

**Published:** 2022-06-03

**Authors:** Lily McCarthy, Theodore C. Hannah, Adam Y. Li, Alexander J. Schupper, Eugene Hrabarchuk, Roshini Kalagara, Muhammad Ali, Alex Gometz, Mark R. Lovell, Tanvir F. Choudhri

**Affiliations:** 1 Department of Neurosurgery, Icahn School of Medicine at Mount Sinai, New York, NY USA; 2 Concussion Management of New York, New York, NY USA; 3 Department of Neurology, University of Pittsburgh, Pittsburgh, PA USA

## Abstract

**Objective/background:**

Despite the prevalence of concussions in young athletes, the impact of headaches on neurocognitive function at baseline is poorly understood. We analyze the effects of a history of headache treatment on baseline ImPACT composite scores in young athletes.

**Methods:**

A total of 11,563 baseline ImPACT tests taken by 7,453 student-athletes ages 12-22 between 2009 and 2019 were reviewed. The first baseline test was included. There were 960 subjects who reported a history of treatment for headache and/or migraine (HA) and 5,715 controls (CT). The HA cohort included all subjects who self-reported a history of treatment for migraine or other type of headache on the standardized questionnaire. Chi-squared tests were used to compare demographic differences. Univariate and multivariate regression analyses were used to assess differences in baseline composite scores between cohorts while controlling for demographic differences and symptom burden.

**Results:**

Unadjusted analyses demonstrated that HA was associated with increased symptoms (β=2.30, 95% CI: 2.18-2.41, *p*<.0001), decreased visual memory (β=-1.35, 95% CI: -2.62 to -0.43, *p*=.004), and increased visual motor speed (β=0.71, 95% CI: 0.23-1.19, *p*=.004) composite scores. Baseline scores for verbal memory, reaction time, and impulse control were not significantly different between cohorts. Adjusted analyses demonstrated similar results with HA patients having greater symptom burden (β=1.40, 95% CI: 1.10-1.70, *p*<.0001), lower visual memory (β=-1.25, 95% CI: -2.22 to -0.27, *p*=.01), and enhanced visual motor speed (β=0.60, 95% CI: 0.11-1.10, *p*=.02) scores.

**Conclusion:**

HA affected symptom, visual motor speed, and visual memory ImPACT composite scores. Visual memory scores and symptom burden were significantly worse in the HA group while visual motor speed scores were better, which may have been due to higher stimulant use in the HA group. The effects of HA on visual motor speed and visual memory scores were independent of the effects of the increased symptom burden.

**Supplementary Information:**

The online version contains supplementary material available at 10.1186/s10194-022-01432-w.

## Introduction

Sports-related concussions (SRCs) in young athletes have become a troubling public health issue. With over 7 million adolescents participating in high school sports annually, sports-related injuries have become progressively more widespread [1]. From 2010-2016, an average of over 280,000 children were treated for SRC or recreation-related traumatic brain injury annually [2]. Approximately 2.5 million high school students reported sustaining one or more SRC in 2017 [3]. The pervasiveness of SRCs in the adolescent population has prompted increased focus on symptoms that occur as a result of these injuries and on the recovery process. An understudied area within concussion research is the relationship between headache history and SRC-related symptoms. To date, only a few studies have investigated whether headache history can predict severity of symptoms, length of recovery, and performance on concussion assessment tools.

Literature to date has indicated that a history of headache (including but not limited to migraine) impact neurocognitive status and symptom burden both before and after SRCs. Adolescent athletes with personal [4] and family [5] migraine histories and headache histories perform differently on neurocognitive evaluations [6–8]. Pre-injury migraine history has been correlated with longer recoveries post-concussion [9]. Pre-injury migraine disorders have been shown to put adolescents at greater risk for higher overall symptom burden and worse cognitive function in the memory domain in the first 72 hours after SRCs [10]. Family history of migraine can also increase the odds of developing post-concussion symptoms even several months after the initial injury [5]. Other studies have similarly reported that family history of migraine disorders is predictive of postconcussion syndrome after SRCs [11]. Thus, there is increasing evidence that a prior history of headache and migraine worsens symptoms, lengthens recovery time, and decreases neurocognitive function after concussion [12]. Importantly, however, others have not discovered any such links between headache history and protracted concussion recovery, which has made it difficult to come to a consensus about the influence of this variable on concussion metrics [13–17].

Although the studies summarized above have all reported differences, there are also a range of null findings in the literature. One study used a series of chi-squared tests to determine whether preconcussion history of migraine or headache influenced protracted recovery (>14 days) versus short recovery (≤14 days) following SRC but did not find that it had any statistically significant effects on length of recovery [13]. Another study primarily focused on the predictive power of preinjury and acute postinjury psychosocial and injury-related variables likewise found no significant correlation between headache history (including both migraine and nonmigraine headaches) and duration of symptom recovery after SRC [14]. A history of migraines was not associated with any symptoms that persisted for more than 28 days in a separate study on predictors of delayed recovery in the aftermath of SRC, further suggesting that this variable may not necessarily result in longer recovery times [15]. Similarly, no relationship between prior migraine history and extended recovery was detected in a molecular study on specific promoter polymorphisms thought to contribute to longer concussion recovery periods in SRC [16]. To that end, it remains to be determined whether a history of headache and migraine is a substantial risk factor for more extended recovery times.

More recently, in addition to the effects of headache and migraine on SRC symptoms and recovery, there has been emerging interest in the effects of a pre-injury history of headache and migraine on baseline scores on neurocognitive assessments. For example, in one study, oculomotor and vestibular examinations of children with premorbid migraines demonstrated these subjects performed worse at baseline than matched controls [18]. However, in another study, baseline scores on Immediate Postconcussion Assessment and Cognitive Testing (ImPACT) in the group with premorbid migraine and control groups did not differ [10]. With such conflicting data, there exists a lack of consensus on baseline performance in this patient population. To that end, this study evaluates the effects of any self-reported premorbid history of headache and migraine treatment on baseline ImPACT composite scores in a large sample of young athletes in order to enhance understanding of neurocognitive function in individuals with pre-existing headache disorders.

## Methods

ImPACT Applications Inc. provided the deidentified data for this study through a research agreement. In total, 11,563 baseline ImPACT tests were provided from assessments performed between 2009 and 2019. There were 7,453 unique subjects ranging in age from 12-22. Only the first baseline test for each of these subjects was included. The headache and/or migraine treatment (HA) cohort was comprised of all individuals who self-reported either i) a history of treatment for headache or ii) a history of treatment for migraine on the standardized questionnaire. Subjects that did not answer were excluded from this study. All other subjects were included in the control (CT) cohort. This study was approved by the Institutional Review Board at the Icahn School of Medicine at Mount Sinai.

ImPACT assesses neurocognitive function in a 20-minute computerized test. Subjects perform various tasks and their performance is graded across multiple composite scores. The composite scores generated by performance on these tasks are verbal memory, visual memory, visual motor speed, and reaction time, and impulse control. Additionally, prior to testing, the subjects complete the Post-Concussion Symptom Scale (PCSS), grading the severity of their symptoms from 0-6 on 22 symptoms which is reported along with the other composite scores. The symptoms that comprise the PCSS are: headache, vomiting, nausea, balance problems, dizziness, trouble falling asleep, sensitivity to light, drowsiness, sensitivity to noise, numbness, fogginess, feeling slowed down, difficulty concentrating, difficulty remembering, visual problems, fatigue, sleeping more than usual, sleeping less than usual, irritability, nervousness, sadness, and feeling more emotional than usual. Importantly, for this study, the PCSS outcome was modified to exclude symptoms that were found to be highly associated with headache in a 2-factor factor analysis (Table S1). The threshold for exclusion was a factor loading greater than 0.30 as has been used previously [19]. The symptoms that were excluded from the PCSS in addition to headache were vomiting, nausea, balance problems, dizziness, trouble falling asleep, sensitivity to light, drowsiness, sensitivity to noise, numbness, fogginess, feeling slowed down, difficulty concentrating, difficulty remembering, and visual problems. Symptoms included in the modified PCSS (mPCSS) were fatigue, sleeping more than usual, sleeping less than usual, irritability, nervousness, sadness, and feeling more emotional than usual.

Demographic comparisons between cohorts were made with t-tests and chi-squared tests. Comparisons of ImPACT composite scores were made with t-tests. Effect sizes were calculated using Cohen’s d. The cohorts were further stratified in subsequent analyses. First the cohorts were subdivided by gender: male and female. Then they were subdivided into three age groupings: 12-14, 15-16, and 17 and older. Finally, they were subdivided into four prior concussion groupings: 0 prior concussions, 1 prior, 2 prior and 3 or more prior. Two-way ANOVAs with post-hoc Tukey’s tests were used to compare composite score outcomes across these groupings. Multivariable regression analyses were used to isolate the effect of prior treatment for headache and/or migraines on baseline neurocognitive performance. The other variables included in the regressions were gender, age, attention-deficit and hyperactivity disorder (ADHD), sport (football versus not football), diagnosed learning disability (DLD), history of concussion, history of psychiatric illness, current selective serotonin reuptake inhibitor (SSRI) use, and current stimulant use. For regression analyses for composite scores other than PCSS, the mPCSS was included as a covariate. Finally, the HA cohort was divided into those with prior treatment for migraine headaches versus those reporting prior treatment for non-migraine headaches and identical regression analyses were performed to evaluate for differences between these two groups. Software used for statistical analyses was Graphpad Prism 9 (San Diego CA). For all analyses, α = 0.05.

## Results

The age of HA and CT groups were similar (15.05±1.59 years vs. 14.95±1.58 years, *p*=.09), and the percentage of male and female patients in each cohort did not significantly differ (37.1% female/62.9% male vs. 34.0% female/66.0% male, *p*=.06). The percentage of individuals who played football also did not significantly differ (41.3% vs. 41.1%, *p*=.91). DLD, ADHD, history of concussion (defined as 2 or more previous concussions), depression/anxiety, SSRI use, and stimulant use were all more common in the HA group (Table 1).

**Table 1:** Cohort Demographics

	CT (*n*=5715) n (%)	Headache/Migraine (*n*=960) n (%)	*p*-value
Age (mean, SD)	14.95 (1.58)	15.05 (1.59)	.09
Sex: Female	1942 (34.0%)	356 (37.1%)	.06
Sport: Football	2352 (41.1%)	397 (41.3%)	.91
DLD	149 (2.61%)	37 (3.85%)	**.03**
ADHD	252 (4.41%)	60 (6.25%)	**.01**
History of Concussion (2 or more)	300 (5.25%)	144 (15.0%)	**<.0001**
Depression/Anxiety	160 {2.80%)	89 (9.27%)	**<.0001**
Psychiatric Medication Use	23 (.40%)	16 (1.67%)	**<.0001**
Stimulant Use	88 (1.54%)	29 (3.02%)	**.001**
BASELINE SCORES	mean [95% CI]	mean [95% CI]	*p*-value
Modified PCSS	2.29 [2.19-2.40]	4.0 [3.67-4.37]	**<.0001**
Verbal Memory	81.66 [81.39-81.93]	81.09 [80.41-81.77]	.12
Visual Memory	70.73 [70.38-71.07]	69.38 [68.51-70.25]	.**004**
Visual Motor	33.90 [33.72-34.08]	34.61 [34.16-35.06]	**.004**
Reaction Time	.640 [.638-.643]	.641 [.635-.647]	.90
Impulse Control	7.11 [6.98-7.24]	7.17 [6.86-7.49]	.73

*CI* Confidence interval

*mPCSS* Modified Post Concussion Symptom Scale

**Fig. 1:**
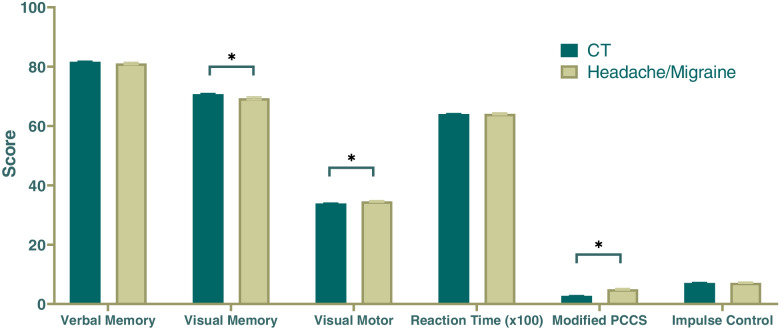
Subjects with a history of treatment for headache and/or migraine had significantly decreased visual memory scores, increased visual motor scores, and greater symptom burden compared to controls. Asterisks indicate significant results

Several composite scores differed significantly between HA and CT. Unadjusted analyses revealed differences in the HA versus CT (Fig. 1; Table 2). These differences were further validated with multivariate analysis, which showed that HA was associated with increased mPCSS at baseline (β=1.40, 95% CI: 1.10 to 2.41, *p*<.0001). HA performed worse in visual memory (β=1.25, 95% CI: -2.22 to -0.27, p=.01) and better in visual motor speed (β=0.60, 95% CI: 0.11 to 1.10, p=.02). In agreement with unadjusted analyses, verbal memory (β=-0.52, 95% CI: -1.28 to 0.24, *p*=.18), reaction time (β=0.001, 95% CI: -0.006 to 0.008, *p*=.77), and impulse control (β=-0.07, 95% CI: -0.43 to 0.29, *p*=.69) scores did not differ significantly between the two groups (Table 1).

**Table 2:** Multivariable analysis of the effect of Headache and Migraine Treatment on Baseline Neurocognitive Function

**UNIVARIATE ANALYSIS**			
**Composite Score**	**Estimate**	**95% CI**	***p*-value**
Modified PCSS	β = 2.30	2.18-2.41	<.0001
Verbal Memory	β = -0.57	-1.28 to 0.15	.12
Visual Memory	β = -1.35	-2.62 to -0.43	.004
Visual Motor	β = 0.71	0.23-1.19	.004
Reaction Time	β = 0.000	-0.007 to .0007	.90
Impulse Control	β = 0.06	-0.28 to 0.40	.73
**MULTIVARIABLE ANALYSIS**			
**Composite Score**	**Estimate**	**95% CI**	***p*-value**
Modified PCSS	β = 1.40	1.10-1.70	<.0001
Verbal Memory	β = -0.52	-1.28 to 0.24	.18
Visual Memory	β = -1.25	-2.22 to -0.27	.01
Visual Motor	β = 0.60	0.11 to 1.10	.02
Reaction Time	β = 0.001	-0.006 to 0.008	.77
Impulse Control	β = -0.07	-0.43 to 0.29	.69

When stratified by gender (Table 3), subjects with a history of treatment for headache and/or migraine had greater symptom burden for both males (CT: 1.78 vs HA: 3.38, *p*<.0001) and females (CT: 3.30 vs HA: 5.10, *p*<.0001). The differences in visual motor speed and visual memory did not reach statistical significance for either males or females. Effect sizes were very small for all composite scores other than mPCSS ranging from 0.002 to 0.11. The effect size of mPCSS analyses was relatively larger, but still small with a Cohen’s d of 0.39 for males and 0.31 for females.

**Table 3:** Effect of HA Treatment on ImPACT Composite Scores by Gender, Age, and Concussion History

**Gender**	**Male**			
	CT (*n*=3773) mean [95% CI]	Headaches (*n*=604) mean [95% CI]	*p*-value	Cohen’s d
Verbal Memory	81.1 [80.8-81.4]	80.6 [79.7-81.4]	.68	0.05
Visual Memory	71.1 [70.6-71.5]	69.8 [68.7-70.9]	.16	0.09
Visual Motor	33.2 [33.0-33.4]	33.9 [33.3-34.5]	.13	0.09
Reaction Time	0.642 [0.639-0.646]	0.645 [0.638-0.653]	.92	0.03
Impulse Control	7.32 [7.16-7.49]	7.46 [7.05-7.87]	.93	0.03
mPCSS	1.78 [1.67-1.88]	3.38 [3.00-3.75]	<.0001	0.39
	**Female**			
	CT (*n*=1942) mean [95% CI]	Headaches (*n*=356) mean [95% CI]	*p*-value	Cohen’s d
Verbal Memory	82.7 [82.3-83.2]	81.9 [80.8-83.1]	.55	0.07
Visual Memory	70.0 [69.4-70.6]	68.6 [67.1-70.0]	.23	0.11
Visual Motor	35.2 [34.9-35.5]	35.8 [35.1-36.6]	.43	0.09
Reaction Time	0.637 [0.6352-0.641]	0.633 [0.624-0.643]	.94	0.04
Impulse Control	6.70 [6.50-6.91]	6.69 [6.20-7.18]	.99	0.002
mPCSS	3.30 [3.07-3.53]	5.10 [4.41-5.79]	<.0001	0.31
**AGE**	**12-14**			
	CT (*n*=2470) mean [95% CI]	Headaches (*n*=390) mean [95% CI]	*p*-value	Cohen’s d
Verbal Memory	81.5 [81.1-81.9]	81.2 [80.2-82.3]	.99	0.03
Visual Memory	71.2 [70.7-71.7]	70.0 [68.6-71.3]	.50	0.10
Visual Motor	32.7 [32.4-32.9]	33.1 [32.4-33.8]	.84	0.07
Reaction Time	0.655 [0.651-0.659]	0.657 [0.647-0.666]	.99	0.016
Impulse Control	7.63 [7.43-7.84]	7.77 [7.26-8.29]	.99	0.03
mPCSS	2.04 [1.89-2.19]	3.42 [3.42-4.54]	<.0001	0.41
	**15-16**			
	CT (*n*=2365) mean [95% CI]	Headaches (*n*=411) mean [95% CI]	*p*-value	Cohen’s d
Verbal Memory	81.6 [81.1-82.0]	80.2 [79.2-81.3]	.38	0.13
Visual Memory	69.9 [69.4-70.5]	68.8 [67.4-70.1]	.59	0.08
Visual Motor	34.2 [34.0-34.5]	34.9 [34.2-35.6]	.47	0.09
Reaction Time	0.635 [0.631-0.639]	0.635 [0.626-0.644]	.99	0.006
Impulse Control	6.90 [6.71-7.10]	7.14 [6.66-7.61]	.95	0.05
mPCSS	2.49 [2.32-2.66]	4.13 [3.62-4.64]	<.0001	0.34
	**17+**			
	CT (*n*=880) mean [95% CI]	Headaches (*n*=159) mean [95% CI]	*p*-value	Cohen’s d
Verbal Memory	82.2 [81.5-82.9]	83.0 [81.4-84.7]	.94	0.08
Visual Memory	71.5 [70.6-72.3]	69.5 [67.1-71.9]	.54	0.14
Visual Motor	36.5 [36.0-37.0]	37.5 [36.4-38.7]	.49	0.14
Reaction Time	0.615 [0.608-0.622]	0.616 [0.604-0.629]	.99	0.002
Impulse Control	6.22 [5.91-6.52]	5.79 [5.11-6.48]	.92	0.09
mPCSS	2.49 [2.20-2.79]	3.81 [2.88-4.74]	.006	0.26
**Concussion History**	**0 Prior Concussions**			
	CT (*n*=4607) mean [95% CI]	Headaches (*n*=591) mean [95% CI]	*p*-value	Cohen’s d
Verbal Memory	81.3 [81.0-81.6]	80.9 [80.1-81.8]	.99	0.04
Visual Memory	70.2 [69.8-70.6]	69.0 [67.9-70.1]	.44	0.08
Visual Motor	33.6 [33.4-33.8]	34.6 [34.0-35.1]	.03	0.14
Reaction Time	0.644 [0.641-647]	0.644 [0.636-0.652]	.99	0.002
Impulse Control	7.07 [6.93-7.21]	6.93 [6.54-7.31]	.99	0.03
mPCSS	2.24 [2.12-2.35]	4.38 [3.91-4.84]	<.0001	0.43
	**1 Prior Concussion**			
	CT (*n*=746) mean [95% CI]	Headaches (*n*=221) mean [95% CI]	*p*-value	Cohen’s d
Verbal Memory	82.6 [81.9-83.4]	80.5 [79.0-82.0]	.13	0.20
Visual Memory	72.3 [71.3-73.2]	69.3 [67.4-71.2]	.07	0.22
Visual Motor	35.1 [34.6-35.7]	34.5 [33.6-35.4]	.92	0.10
Reaction Time	0.625 [0.617-0.632]	0.642 [0.631-0.653]	.33	0.19
Impulse Control	7.29 [6.93-7.65]	7.71 [7.01-8.40]	.96	0.08
mPCSS	2.41 [2.10-2.73]	3.23 [2.58-3.89]	.20	0.18
	**2 Prior Concussions**			
	CT (*n*=193) mean [95% CI]	Headaches (*n*=77) mean [95% CI]	*p*-value	Cohen’s d
Verbal Memory	83.8 [82.5-85.2]	84.5 [82.3-86.8]	.99	0.07
Visual Memory	74.5 [72.7-76.2]	70.7 [67.9-73.6]	.43	0.30
Visual Motor	35.5 [34.5-36.5]	34.4 [32.7-36.1]	.95	0.14
Reaction Time	0.638 [0.621-0.655]	0.634 [0.613-0.655]	.99	0.04
Impulse Control	7.20 [6.52-7.87]	6.53 [5.50-7.57]	.98	0.14
mPCSS	2.74 [2.06-3.41]	4.03 [2.83-5.22]	.34	0.26
	**3+ Prior Concussions**			
	CT (*n*=107) mean [95% CI]	Headaches (*n*=67) mean [95% CI]	*p*-value	Cohen’s d
Verbal Memory	83.1 [81.2-85.1]	80.6 [77.9-83.4]	.13	0.23
Visual Memory	75.1 [72.4-77.7]	70.8 [67.6-74.1]	.47	0.31
Visual Motor	34.8 [33.2-36.4]	35.6 [33.6-37.7]	.99	0.10
Reaction Time	0.614 [0.598-0.629]	0.623 [0.602-0.643]	.99	0.11
Impulse Control	8.06 [6.90-9.23]	8.22 [6.77-9.68]	.99	0.03
mPCSS	2.75 [1.89-3.60]	3.73 [2.05-4.69]	.98	0.12

*CI* Confidence interval

*mPCSS* Modified Post Concussion Symptom Scale

When stratified by age (Table 3), the differences in visual memory (12-14: 71.2 vs 70.0, p=.50; 15-16: 69.9 vs 68.8, p=.59; 17+: 71.5 vs 69.5, p=.54) and visual motor speed (12-14: 32.7 vs 70.0, *p*=.84; 15-16: 34.2-24.9, *p*=.47; 17+ 36.5 vs 37.5, *p*=.49) were not seen in any grouping. All three subgroups demonstrated a significant effect of HA on mPCSS (12-14: 2.04 vs 3.42, *p*<.0001; 15-16: 2.49 vs 4.13, *p*<.0001; 17+: 2.49 vs 3.89, *p*=.006). Effect sizes were again very small for all composite scores other than mPCSS ranging from 0.002-0.14. Cohen’s d for mPCSS ranged from 0.26-0.41.

When stratified by history of prior concussion (Table 3), the group with no prior concussions demonstrated similar results, with the HA subjects performing better on visual motor speed (33.6 vs 34.6, *p*=.03) despite higher mPCSS (2.24 vs 4.38, *p*<.0001). The difference in visual memory (70.2 vs 69.0, *p*=.44) and other composite scores did not reach statistical significance. There were no statistical differences in any of the composite scores or symptom burden in subjects with 1-prior, 2-prior or 3+ concussions, although the trend of higher symptom burden and lower visual memory scores remained consistent in each of those groups. Effect sizes for these analyses ranged from 0.002 to 0.43.To evaluate potential differences in neurocognitive performance between participants with migraines versus other types of headaches, the HA group was split into those that reported prior treatment for migraine headaches and those who did not report prior treatment for migraines. Demographic differences are reported in (Table 4). Participants with prior migraine treatment were slightly older (15.2 vs 14.9, *p*=0.01) than those reporting treatment for other headaches. They were also more likely to have a history of psychiatric illness (10.9% vs 6,9%, *p*=0.04). There were no differences between groups in terms of gender (37.2% vs 36.9%, *p*=0.93), percentage playing football (42.1% vs 40.0%,*p*=0.52), ADHD (7.4% vs 4.6%, *p*=0.08), prior concussion history (16.1% vs 13.3%, *p*=0.23), SSRI use (1.8% vs 1.5%, *p*=0.80), or stimulant use (3.3% vs. 1.8%, *p*=0.07).

**Table 4:** Demographics of Subjects with Prior Migraine Treatment vs Treatment for Other Headache Types

	Migraine (*n*=570) n (%)	Other Headache (*n*=390) n (%)	*p*-value
Age (mean, SD)	15.2 (1.7)	14.9 (1.5)	**0.01**
Sex: Female	212 (37.2%)	144 (36.9%)	0.93
Sport: Football	240 (42.1%)	156 (40.0%)	0.52
DLD	25 (4.4%)	12 (3.1%)	0.30
ADHD	42 (7.4%)	18 (4.6%)	0.08
History of Concussion (2 or more)	92 (16.1%)	52 (13.3%)	0.23
History of Psychiatric Illness	62{10.9%)	27 (6.9%)	**0.04**
SSRI Use	10 (1.8%)	56(1.5%)	0.80
Stimulant Use	22 (3.9%)	7 (1.8%)	0.07

In univariate analyses, there were no differences between the groups for any ImPACT composite scores or symptom burden. The multivariable analysis also revealed no difference in performance on any of the composite scores (Table 5).

**Table 5:** Multivariable Linear Regression Analysis of the Effect of Migraine Treatment versus Treatment of Other Headache Types on Baseline Neurocognitive Function

**UNIVARIATE ANALYSIS**			
**Composite Score**	**Estimate**	**95% CI**	***p*-value**
Modified PCSS	β = 0.24	-0.47 to 0.95	0.51
Verbal Memory	β = 0.31	-1.07 to 1.69	0.66
Visual Memory	β = 0.60	-1.18 to 2.37	0.51
Visual Motor	β = 0.77	-0.15 to 1.68	0.10
Reaction Time	β = -0.011	-0.024 to -0.001	0.06
Impulse Control	β = -0.24	-0.89 to 0.40	0.46
**MULTIVARIABLE ANALYSIS**			
**Composite Score**	**Estimate**	**95% CI**	***p*-value**
Modified PCSS	β = 0.18	-0.54 to 0.90	0.62
Verbal Memory	β = 0.04	-1.41 to 1.48	0.96
Visual Memory	β = 0.56	-1.29 to 2.42	0.55
Visual Motor	β = 0.46	-0.46 to 1.37	0.97
Reaction Time	β = -0.009	-0.021 to 0.003	0.14
Impulse Control	β = -0.08	-0.75 to 0.58	0.80

## Discussion

### Overview

In this study, we aimed to determine whether a history of headache and migraine treatment affected baseline ImPACT composite scores. Our results suggest that individuals with this profile demonstrate higher symptom burden, lower visual memory, and higher visual motor scores at baseline. From the results of the current study, the effect of headache and migraine treatment on visual memory was detrimental, while the effect on visual motor speed appeared beneficial. Both findings were independent of the observed increased symptom burden. In contrast, verbal memory, reaction time, and impulse control scores did not significantly differ. Given the dearth of studies on baseline performance in adolescents with preexisting headache history, our results shed much-needed light on neurocognitive characteristics of this previously understudied patient population, providing a foundation for deeper examination of the effects of HA on neurocognitive functioning before and after head injuries.

### mPCSS (Symptom burden)

The HA group had greater symptom burden, with increased mPCSS composite scores. Our finding suggests that caution should be taken when making a decision about diagnosis or treatment based on the changes in ImPACT values that can be suppressed or enhanced while under HA treatments, potentially helping to reveal whether symptoms seen after a concussion are due to the injury itself or preexisting pathologies such as headache [20]. This specific data point is therefore highly clinically valuable because it may prevent clinicians from mistakenly ascribing certain symptoms to concussions [21]. Although relatively little has been published on baseline performance in this specific patient population, a few other studies have reported similar results. For instance, Mannix et al. found that adolescent athletes with a history of medical treatment for headaches reported more baseline symptoms [22]. In fact, in the multivariate model from this same study, headache/migraine history was one of the most common factors related to baseline preseason symptom reporting, second only to mental health history.

Cottle et al. likewise reported that previous treatment for headaches and migraines correlated with increased total symptom score at baseline, as measured in National Collegiate Athletic Association (NCAA) Division I collegiate student-athletes via ImPACT [23]. Register-Mihalik et al. also discovered a similar association between preseason headaches and baseline symptom score/severity in their cohort of high-school and collegiate athletes [6]. Finally, Solomon et al. detected a significant difference between average total symptom scores on ImPACT in headache-treated and non-treated groups of National Football League (NFL) players, with those in the former reporting more symptoms on average [24]. Thus, our observation aligns with those from prior investigations into baseline symptomatology in this patient demographic.

### Visual memory

Visual memory scores were also lower in the HA group. Once again, it is difficult to place this observation in context with previously reported findings because so few studies have been conducted on baseline performance in this subgroup. In the same study by Cottle et al., individuals previously treated for headaches had decreased visual memory scores on ImPACT testing; however, these differences were not ultimately statistically significant (71.0 vs. 76.0, d_baseline_ = -5.0, *p* =.048) [23]. In another study by Mihalik et al., visual memory scores in HA and non-HA groups did not significantly differ (75.69 vs. 74.74, d_baseline_ = 0.95) [25]. Terry et al. likewise found no difference in baseline performance for children with premorbid migraines compared to controls, including visual memory scores (70.59 vs. 71.19, d_baseline_ = -0.04) [10]. It should be noted that given the extremely low effect sizes of the differences in Visual Memory scores in the present study, these results may actually be consistent with prior works. Still, additional research is warranted to confirm whether HA individuals perform worse in visual memory or comparably to their non-HA counterparts. Notably, visual memory scores were consistently lower in the HA group even when subdivided into categories based on prior concussion history. Individuals with HA performed worse on this section regardless of whether they had sustained no, one, two, or three concussions. This finding suggests that decreased visual memory may be the common variable associated with HA. In other words, poorer performance on visual memory tasks as measured via ImPACT appears to be more significantly related to headache etiology than to the concussion itself.

### Visual motor speed

In contrast, visual motor speed scores were higher in the HA group, a surprising trend that has not been widely reported in the literature. In fact, Cottle et al. found that visual motor speed scores were lower in those treated for migraines (*p*=.015), which directly conflicts with our results [23]. Mihalik et al. observed a similar pattern, albeit statistically insignificant (*p* = .054), of reduced visual motor speed scores in HA individuals [25]. Terry et al. did not find any significant differences in visual motor speed scores between migraine and control groups (*p* = .06) [10]. Although the low effect size of our result could signal a lack of clinical relevance and thus be consistent with prior reports, one other potential explanation for our disparate finding is that stimulant use was higher in our HA group. The use of stimulants, which was more prevalent in HA individuals, may have improved their visual motor speed performance on baseline ImPACT testing.

Although the use of stimulants has not been well studied in patients with a history of headaches, there has been some literature published on those with other clinical profiles, including ADHD [26]. Gardner et al. found that young athletes with ADHD and stimulant treatment had higher visual motor speed scores at baseline than those without any such treatment [27]. Even in healthy participants without any such disorders, stimulant use has been shown to improve visual processing speed, leading to increased visual motor speed scores on ImPACT testing [28]. Thus, there are emerging signs that stimulants may alter neurocognitive performance on ImPACT, at least in certain cohorts, which may be a confounder for our findings. Future studies will concentrate on HA individuals and will more closely examine the potential effects of stimulant use on baseline results in this patient population.

### Verbal memory, reaction time, & impulse control

We did not detect any statistically significant differences in verbal memory scores, reaction time, or impulse control in our univariate or multivariable analyses, in line with findings from previous studies [23–25]. Hence, at least based on current evidence, HA individuals do not seem to perform differently than their non-HA counterparts in these areas on baseline ImPACT testing. That said, research on this patient population is still largely in this infancy, and so further exploration is needed to confirm that headache history does not have a meaningful influence on any of these three domains.

### Migraine versus other headache types

This is the first study we are aware of that directly compared baseline performance between those who reported a history of migraine specifically versus those who report a history of unspecified headache. After controlling for appropriate covariates, we found no significant difference between the groups. This work requires future corroboration, but provides confidence that, despite differences in pathogenesis between migraine and other types of headaches, the primary analyses in this project were not unduly affected by combining subjects reporting prior treatment for migraine and those reporting prior treatment for non-specific headache.

### Limitations

Our study involved a retrospective cross-sectional methodology, as have most other studies that have assessed different groups of adolescent athletes with baseline ImPACT testing. In order to attain a more comprehensive sense of baseline neurocognitive functions and how they might evolve, incorporating longitudinal schemes would be valuable. Very few have explored this avenue of investigation. However, one recent study evaluated the test-retest reliability of memory and speed of ImPACT over the course of two years across several different groups, including one with a history of treatment for headache/migraine [29]. These findings demonstrate the feasibility and utility of conducting baseline testing at multiple time points, especially in individuals with HA and other premorbid conditions. Future studies should strive to monitor baseline performance by building in more of these longitudinal designs. Additionally, prospective investigations should explore variations in different areas the HA group might have in the absence of ImPACT with new modalities such as eye tracking technology.

Utilizing other test batteries that more thoroughly assess neurocognitive and neuropsychological functions might also be worthwhile, helping to clarify whether HA results in deficits not visible on a short, computerized test such as ImPACT. Even though our findings increase confidence in evaluations of individuals with head injury, variations of responses are common, and other methods of assessment should be considered before making a diagnosis or treatment. Conducting additional studies, including ones that involve magnetic resonance imaging (MRI) or even functional magnetic resonance imaging (fMRI), would also help to correlate results observed here with specific imaging findings. fMRI has been used to probe the structural basis for conditions such as migraine, which is known to cause sensory hypersensitivities, pain, and other debilitating neurological complaints [30–32]. However, neither MRI nor fMRI has been used extensively in studies on adolescent athletes with this pathology or other types of headaches. These brain imaging modalities could help to uncover the anatomical and functional foundations for neurocognitive differences registered on tests such as ImPACT.

The higher prevalence of stimulant use in our HA group may have also skewed our data, leading to higher visual motor speed scores. Clarifying the role of stimulants with future work will help to address this concern. Additionally, for those subjects reporting non-migraine ‘headaches’, we do not know what type of headache they experienced. Other potential shortcomings of our study include the fact that we did not incorporate independent validity or effort indicators apart from those in ImPACT. We were also unable to validate certain self-reported information, including SSRI use and history of previous concussion. However, there is growing evidence that adolescent athletes report clinical details such as concussion history with a high degree of reliability, making this feature of our study slightly less objectionable [33]. Finally, we were not able to address the possibility that the certain subjects may have taken previous ImPACT tests prior to this study, which could have contributed to baseline differences observed.

## Conclusion

The results from this study suggest that prior treatment for headache or migraine affects baseline performance on ImPACT. HA was associated with greater mPCSS, lower visual memory scores, and higher visual motor speed scores. HA had a negative effect on visual memory but a positive influence on visual motor scores, which may be a function of the increased stimulant use observed in this group. Knowledge of these baseline differences in individuals with headache histories may help to guide patient care both pre- and post-concussions. Given the low effect sizes of results, further work is necessary to confirm the clinical relevance of these findings.

## Supplementary Information

**Additional file 1: Table S1.** Factor Analysis of Symptoms.

## Data Availability

The dataset(s) supporting the conclusions of this article will be available upon request.

## Abbreviations

ADHD: Attention deficit and hyperactivity disorder
CT: Control
DLD: Diagnosed learning disability
HA: Headache and/or migraine
ImPACT: Immediate Postconcussion Assessment and Cognitive Testing
mPCSS: Modified PCSS
SRC: Sports-related concussions
SSRI: Selective serotonin reuptake inhibitor
