# Supplementary material for: Effects of a history of headache and migraine treatment on baseline neurocognitive function in young athletes
Source: J Headache Pain. 2022 Jun 3;23(1):62. doi: 10.1186/s10194-022-01432-w (PMC9164363; doi:10.1186/s10194-022-01432-w)
Supplement: Supplementary file 1 — Additional file 1: Table S1. Factor Analysis of Symptoms. [file 10194_2022_1432_MOESM1_ESM.docx]

**Table S1 Factor Analysis of Symptoms.** To create the modified Post-Concussion Symptom Scale (mPCSS) a two-factor structure was created. Headache was found to have a factor loading >0.30 in Factor 2. Thus, symptoms in Factor 2 with a factor loading >0.30 were considered significantly related to headache and excluded from the mPCSS.

| **Symptom** | **Factor 1** | **Factor 2** | **Exclude from mPCSS?** |
| --- | --- | --- | --- |
| Headache | 0.284792 | **0.544887** | yes |
| Vomiting | 0.097164 | **0.442866** | yes |
| Nausea | 0.191672 | **0.558665** | yes |
| Balance Problems | 0.206264 | **0.627167** | yes |
| Dizziness | 0.197258 | **0.721377** | yes |
| Trouble Falling Asleep | 0.479354 | **0.315335** | yes |
| Fatigue | 0.38093 | 0.298263 | no |
| Sleeping More than Usual | 0.224954 | 0.263844 | no |
| Sleeping Less than Usual | 0.485425 | 0.225883 | no |
| Sensitivity to Light | 0.335728 | **0.406654** | yes |
| Drowsiness | 0.417637 | **0.329184** | yes |
| Sensitivity to Noise | 0.372162 | **0.377222** | yes |
| Irritability | 0.538148 | 0.236925 | no |
| Nervousness | 0.582135 | 0.21756 | no |
| Sadness | 0.720521 | 0.127976 | no |
| Feeling More Emotional | 0.743068 | 0.109989 | no |
| Numbness | 0.286816 | **0.450173** | yes |
| Fogginess | 0.451883 | **0.344244** | yes |
| Feeling Slowed Down | 0.458264 | **0.354905** | yes |
| Difficulty Concentrating | 0.537313 | **0.373172** | yes |
| Difficulty Remembering | 0.388081 | **0.336741** | yes |
| Visual Problems | 0.307601 | **0.30537** | yes |
